# Time‐Varying Spatial Propagation of Brain Networks in fMRI Data

**DOI:** 10.1002/hbm.70131

**Published:** 2025-01-21

**Authors:** Biozid Bostami, Noah Lewis, Oktay Agcaoglu, Jessica A. Turner, Theo van Erp, Judith M. Ford, Mahshid Fouladivanda, Vince Calhoun, Armin Iraji

**Affiliations:** ^1^ School of Electrical and Computer Engineering Georgia Institute of Technology Atlanta Georgia USA; ^2^ Tri‐Institutional Center for Translational Research in Neuroimaging and Data Science (TReNDS) Georgia State, Georgia Tech, and Emory Atlanta Georgia USA; ^3^ School of Computational Science and Engineering Georgia Institute of Technology Atlanta Georgia USA; ^4^ Department of Psychiatry and Behavioral Health University of California Irvine California USA; ^5^ School of Medicine University of California Irvine California USA; ^6^ Department of Psychiatry University of California San Francisco California USA; ^7^ Department of Computer Science Georgia State Atlanta Georgia USA

**Keywords:** dynamic states, network propagation, resting‐state fMRI, sliding window, spatial dynamic propagation

## Abstract

Spontaneous neural activity coherently relays information across the brain. Several efforts have been made to understand how spontaneous neural activity evolves at the macro‐scale level as measured by resting‐state functional magnetic resonance imaging (rsfMRI). Previous studies observe the global patterns and flow of information in rsfMRI using methods such as sliding window or temporal lags. However, to our knowledge, no studies have examined spatial propagation patterns evolving with time across multiple overlapping 4D networks. Here, we propose a novel approach to study how dynamic states of the brain networks spatially propagate and evaluate whether these propagating states contain information relevant to mental illness. We implement a lagged windowed correlation approach to capture voxel‐wise network‐specific spatial propagation patterns in dynamic states. Results show systematic spatial state changes over time, which we confirmed are replicable across multiple scan sessions using human connectome project data. We observe networks varying in propagation speed; for example, the default mode network (DMN) propagates slowly and remains positively correlated with blood oxygenation level‐dependent (BOLD) signal for 6–8 s, whereas the visual network propagates much quicker. We also show that summaries of network‐specific propagative patterns are linked to schizophrenia. More specifically, we find significant group differences in multiple dynamic parameters between patients with schizophrenia and controls within four large‐scale networks: default mode, temporal lobe, subcortical, and visual network. Individuals with schizophrenia spend more time in certain propagating states. In summary, this study introduces a promising general approach to exploring the spatial propagation in dynamic states of brain networks and their associated complexity and reveals novel insights into the neurobiology of schizophrenia.

## Graphical abstract

In this work, we proposed a novel methodology for tracking propagation of a specific brain network utilizing lagged, sliding window Pearson correlation to capture voxel‐level network propagation that evolves with time.

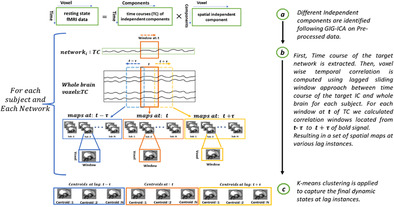

## 1 Introduction

Spontaneous neural activity in the human brain can occur in the absence of external stimuli and be observed at different spatial and temporal scales (Kucyi et al. 2018). The neural activity throughout the cortex has been studied via various imaging modalities, such as calcium imaging (Ikegaya et al. 2004; Stroh et al. 2013) and voltage‐sensitive dye (Petersen et al. 2003). For noninvasive, macro‐scale functional neuroimaging studies, resting‐state functional magnetic resonance imaging (rsfMRI) is used to understand and study intrinsic brain activity (Fox and Raichle 2007). The standard approach computes temporal coupling between the blood oxygenation level‐dependent (BOLD) signals, also known as functional connectivity (Biswal et al. 1995). Such studies were then extended to estimate multiple functional networks spanning the brain, including the visual and default mode networks (DMN) (Buckner et al. 2011). Among the various methodologies, independent component analysis (ICA) is a well‐known approach that extracts spatial patterns of different brain networks (i.e., functional connectivity maps) and their associated temporal activity (McKeown, Hansen, and Sejnowski 2003; Calhoun, Kiehl, and Pearlson 2008).

Understanding the nature of spontaneous neural activity is an active area of research. Introducing temporal lags to capture whole‐brain lag structures and representing patterns that shift over time has been studied in the context of region of interest analyses (Mitra et al. 2015). Other approaches have focused on capturing a set of global recurring consecutive activity patterns, such as quasiperiodic patterns, highlighting propagation across areas of the task‐positive and the DMN (Abbas, Bassil, and Keilholz 2019; Xu et al. 2023). Global coactivation patterns, for example, transient neuronal coactivation patterns, can also be related to globally propagating waves (Majeed, Magnuson, and Keilholz 2009; Liu and Duyn 2013), with most focusing only on signal peaks, while other approaches focus on continuously varying contributions (Iraji et al. 2022). Other studies show the ability to capture time‐varying brain connectivity, which can be helpful in diagnosing disorders such as schizophrenia (SZ) (Calhoun et al. 2014; Miller et al. 2022; Miller, Pearlson, and Calhoun 2019). Studies of whole‐brain dynamic connectivity also focus on the temporal coupling within and between the functional domains (Allen et al. 2014; Damaraju et al. 2014). Other work has specifically evaluated spatial brain networks using a sliding window approach (Kiviniemi et al. 2011). This work suggests that the fMRI data captures moment‐to‐moment voxel‐wise changes within functional brain networks, such as the DMN. However, previous studies ignore the spatial fluidity of the brain networks which evolve with time. Spatial fluidity can be defined as the transitory spatial pattern of a given functional network over time at the voxel‐level measurement (Iraji et al. 2019).

To our knowledge, there is no work focused on quantifying propagating spatial patterns in fMRI data within spatial dynamic states (i.e., substates voxel‐wise dynamics within specific brain networks) (Matsui, Murakami, and Ohki 2016). Here, we focus on understanding network‐specific dynamic spatial state propagation. This study presents an approach to estimating multiple brain networks using ICA and capturing time‐varying propagation using lagged windowed correlations and spatial dynamic state analysis. We first evaluate the replicability of the results, followed by a study focused on changes linked to mental illness. Results show clear evidence that we can detect replicable evolution of brain networks over time, such as default mode, visual, and temporal networks. Important propagation properties, such as propagation speed and pattern show variation across dynamic states. Results also show that these spatially propagating patterns are linked to mental illness. For example, studied networks showed that subjects who are diagnosed with SZ have higher dwell time compared to the control group, suggesting that subjects diagnosed with SZ have less activity in their brain networks and remain dominant in a particular dynamic state. This study is a first step toward understanding the complex nature of network‐specific spatial dynamic propagation.

## 2 Materials and Methods

### 2.1 Data Information and Preprocessing

In this study, we used resting‐state eyes‐closed fMRI data. One of the challenges of eyes closed is individuals may sleep more easily. In our case, individuals did indicate that they were awake in post‐scan interviews conducted on a random subset of subjects. While this is not definitive, it does provide confidence that they were following instructions.

For the data, two different datasets are used. The first dataset is from the Human Connectome Project for Early Psychosis (HCP‐EP) (Human Coonectome Dataset n.d.). The second dataset is the Functional Imaging Biomedical Informatics Research Network (FBIRN) (Keator et al. 2016). The HCP‐EP dataset is used to validate our proposed method and evaluate replicability across two scan sessions. We select a subsection of the HCP‐EP dataset where subjects are common across the two scan sessions. To evaluate the replicability of our proposed method, we used the data from the two scan sessions and matched the results after running the pipeline independently. The FBIRN dataset will be our primary dataset for evaluating links to clinical diagnosis and result analysis.

In the HCP‐EP dataset, there were 163 subjects in each session remaining after preprocessing and quality control were done. The data were collected from 4 clinical recruitment sites and the consent form of each participant was collected before scanning. Medically stable male and female subjects with a confirmed psychiatric diagnosis and healthy control (HC) subjects were enrolled in the HCP‐EP study. The image data were scanned using Siemens MAGNETOM Prisma 3T scanners with a multiband sequence and a 32/64‐channel head coil. The rs‐fMRI data had 2‐mm isotropic resolution, multiband acceleration factor of 8, repetition time (TR) = 720 ms, and was acquired twice with posterior–anterior (PA) and anterior–posterior (AP) phase encoding. More details about the dataset can be found on the official website of the National Institutes of Health (Human Coonectome Dataset n.d.).

The fMRI data were preprocessed using a combination of FSL and statistical parametric mapping (SPM12) under the MATLAB 2020 environment. Before motion correction, a distortion field was calculated from the PA and AP phase‐encoded field maps by the top‐up/FSL algorithm to correct for intensity and geometric distortions. Then, a rigid body motion correction was performed using SPM to fix the head motions in fMRI scans. After that, the fMRI data were normalized to the standard Montreal Neurological Institute (MNI) space using an echo‐planar imaging (EPI) template and slightly resampled to 3 × 3 × 3 mm^3^ isotropic voxels. The resampled fMRI images were smoothed using a Gaussian kernel with a full‐width at half‐maximum (FWHM) = 6 mm. Since dynamic functional connectivity analysis can be sensitive to the data quality, we performed quality control (QC). We selected subjects with functional data providing near‐full brain successful normalization for further analysis. This yielded a total of 170 subjects. More details can be found in the following study (Fu, Iraji, Sui, et al. 2021).

The second dataset for evaluating links to clinical diagnosis was fBIRN (Keator et al. 2016). The groups consisted of 160 typical controls with a mean age of 36.9 years and 150 individuals with SZ with a mean age of 37.8 years. There were 115 control males and 114 males with SZ. Also, 45 female controls and 36 females with SZ. Seven sites across the United States collected eyes‐closed rsfMRI data. Consent forms were collected following the regulation of Internal review boards of the affiliated institutions prior to scanning. Six sites used the Siemens Tim Trio System, and one used the General Electric Discovery MR750 scanner. Resting‐state fMRI scans were acquired following a standard gradient‐echo EPI paradigm: Field of view (FOV) of 220 × 220 mm^2^ (64 × 64 matrices^2^), TR = 2000 ms, TE = 30 ms, FA = 770°, 162 volumes, 32 sequential ascending axial slices of 4 mm thickness, and 1 mm skip. Data preprocessing used a combination of various toolboxes, such as AFNI (Cox 1996), SPM (SPM12 n.d.), and GIFT (Gift Toolbox for ICA n.d.). We used the INRIAlign toolbox to correct head motion in SPM. 3dDespike algorithm from AFNI was applied to remove outliers. Then, fMRI data were resampled to 3 mm^3^ isotropic voxels. Then, data were smoothed to 6 mm FWHM using the BlurToFWHM algorithm of the AFNI toolbox, and each voxel time course was variance normalized.

### 2.2 Independent Component Analysis

ICA is one of the most common approaches for blind source separation. It is based on the assumption that any captured signal (x) can be defined as a linear combination (A) of its latent sources (s), which are mutually independent such that x=As (Calhoun et al. 2001), where x is a vector representing the captured mixtures, represents latent sources, and A is a mixing matrix where $A\in \mathbb{R}\;\left(N\times M\right)$. ICA aims to estimate an unmixing matrix called $W\in \mathbb{R}\;\left(N\times M\right)$ such that y=Wx, which approximates the latent sources (s) subject to permutation and scaling ambiguities. Implementation of ICA was via the GIFT toolbox (http://trendscenter.org/software/gift). In practice, M>N, so dimension reduction is first applied. First subject‐level spatial principal component analysis (PCA) was applied, and 99% of the subject‐level variance was retained. Next, group‐level spatial PCA was applied on concatenated subject‐level principal components (PCs) for all subjects. Twenty group‐level PCs were selected for future analysis, which is sufficient to capture the standard large‐scale resting networks. Next, Infomax ICA was applied to estimate 20 maximal ICs. Infomax was repeated 100 times, and the ICASSO framework was used to select the best (most central) component run to ensure the stability and reliability of the ICs (Calhoun et al. 2001). Subject‐specific ICs and associated time courses were derived using spatially constrained ICA, group information‐guided ICA using the group map as the reference (Du and Fan 2013). Finally, different brain networks, such as the DMN, visual, temporal lobe, and subcortical (SC) network were identified based on their spatial maps and power spectra (Allen et al. 2011). The selected network information is mentioned in Table 1.

**TABLE 1:** Selected components and their information.

Network	Peak value	Coordinate's
Default mode network (Comp 13)		
Precuneus right	12.10	27, 17, 35
Posterior cingulate gyrus right	10.01	27, 27, 35
Dorsal anterior cingulate gyrus	8.41	27, 30, 37
Prefrontal cortex	3.57	27, 57, 22
Rostral anterior cingulate gyrus	6.14	43, 22, 35
Angular gyrus right	8.96	42, 18, 36
Angular gyrus left	8.27	13, 17, 36
Subcortical network (Comp 14)		
Putamen right	15.60	35, 40, 25
Putamen left	16.25	19, 40, 35
Visual Network (Comp 16)		
Lingual gyrus left	11.56	22, 12, 20
Cuneus right	9.85	27, 11, 26
Fusiform gyrus right	9.57	32, 37, 38
Temporal network (Comp 4)		
Postcentral gyrus left	11.3	46, 38, 32
Postcentral gyrus right	10.7	8, 38, 33
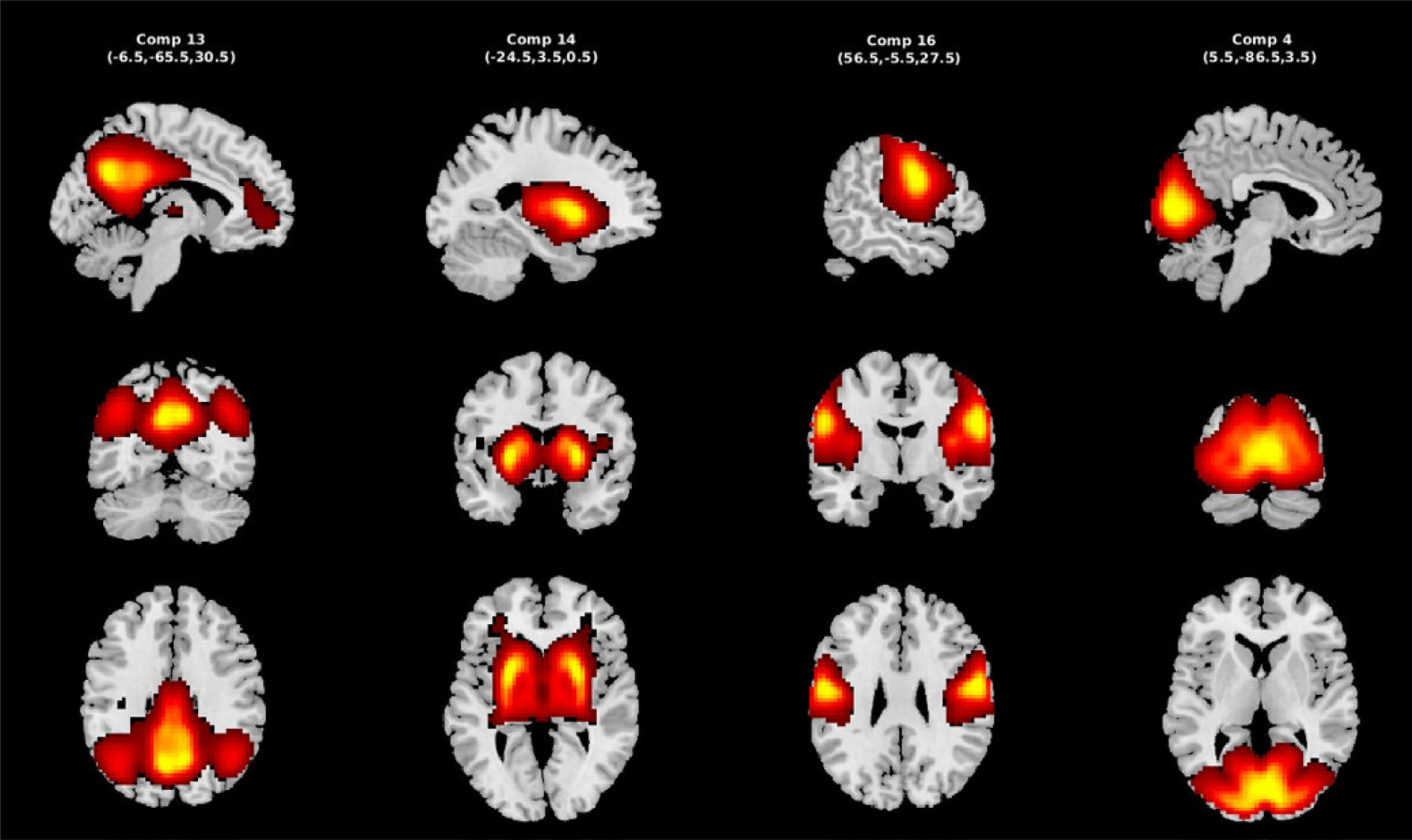		

### 2.3 Calculating Spatial Maps With Lagged Window Correlation

The dynamic spatial propagation of various brain networks can be evaluated at a voxel‐wise level. For this purpose, the temporal coupling between the selected brain network and every brain voxel using the lagged sliding window approach was calculated. The same cleaning procedures were followed, effectively capturing dynamic patterns on time courses and every brain voxel to reduce noise (Damaraju et al. 2014). The cleaning procedure included orthogonalizing to estimated subject motion parameters, linear detrending, despiking, and bandpass filtering using a fifth‐order Butterworth filter (0.01–0.15 Hz). The tapered window was obtained by convolving a rectangle (width = 30 TRs: with a Gaussian) (σ = 3 TRs), and the sliding step size of 2 TR was used. In this study, a parameter, lag (τ) was introduced, to indicate the temporal correlation between a given brain network and every voxel of the brain for every (t±τ).

As the sliding window shifts along the time course of the target network, temporal correlation between the windowed time points located at time t of the selected ICA time course and the windowed time points located at (t±τ) for every value of τ is calculated. In more general terms, multiple voxel‐level temporal correlations by shifting the window over bold time signals for different τ values are computed while keeping the ICA network's window location fixed.

For example, if τ=2andTR=2 s is selected, then a set of 5 spatial maps located at (−4, −2, 0, 2, and 4 s) for every sliding step t will be captured. These are called “lag points”. More generally, if τ=n, the 2n+1 spatial maps are computed for each window step. Each window's correlation variation at different times was captured by introducing the lag parameter.

### 2.4 Calculation of Dynamic Spatial States

**FIGURE 1:**
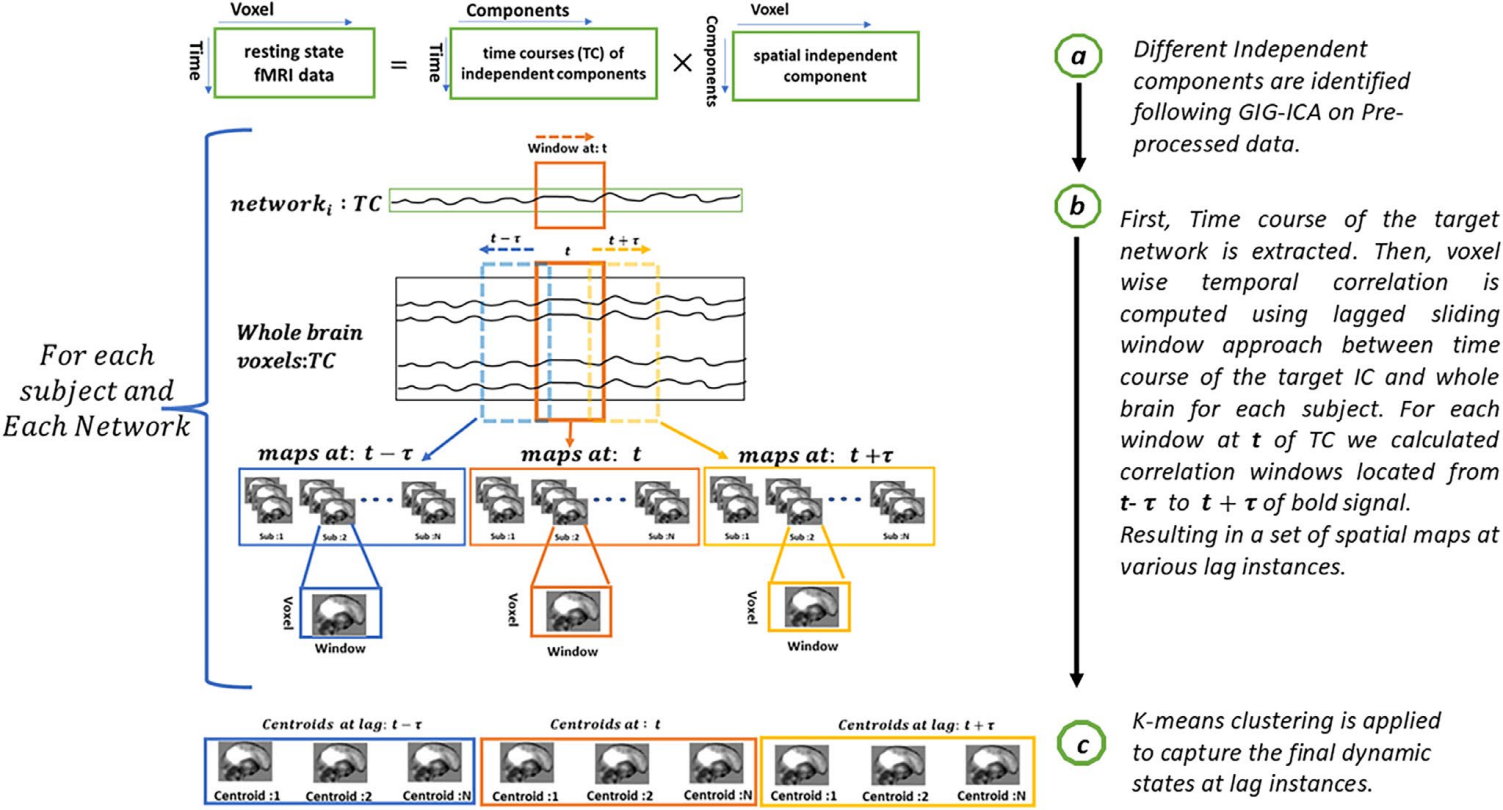
Analysis pipeline of the proposed lagged windowed correlation approach. The proposed pipeline uses a lagged sliding window approach to capture voxel‐level network propagation that evolves with time.

For each resting‐state network, after calculating the lagged spatial maps for every subject, a three‐dimensional matrix was created with dimensions representing as follows: (voxel × lag × window). Then, this matrix was flattened along the first two dimensions, resulting in a two‐dimensional matrix (voxel*lag × window). Next, concatenated all subject's lagged windowed spatial maps (voxel*lag × window*#subject). Then K‐means clustering was applied to this matrix to find the dynamic states (voxel*lag × 1). For each cluster, the spatial maps at each lag point were retrieved. k‐means clustering was used to identify the spatial dynamic states and associated patterns. For this study, the cluster number of 4 was selected, the determination of the optimal cluster number was determined with the Elbow criteria within the GIFT software (Gift Toolbox for ICA n.d.), this is also consistent with the prior clustering work (Fu, Iraji, Turner, et al. 2021). The clustering was replicated 50 times with different initializations using the k‐means++ method to increase the probability of avoiding local minima (Arthur and Vassilvitskii 2007). The correlation distance was used as the metric to calculate the similarity between the data points. The analysis of dynamic states was done in two steps. First, ran k‐means clustering for each subject and captured exemplar (or reference) states. Next, run a second level of k‐mean, merging all the exemplar states to obtain exemplar centroids. These centroids are used as a reference for calculating the final centroids, computed by concatenating all subject's spatial maps and running k‐means clustering. The complete pipeline is shown in Figure 1.

### 2.5 Statistical Experiments

A number of summary measures were calculated on the output, including mean dwell time (MDT), fraction time (FT), and disperse rate (DR). MDT calculates the average time spent at each state before transitioning to other states. FT gives the percentage of total time in each state. DR represents the distance between spatial maps at different lag points, which tells us how fast the network propagates. We observed group differences based on the calculated features.

## 3 Experiments and Results

**FIGURE 2:**
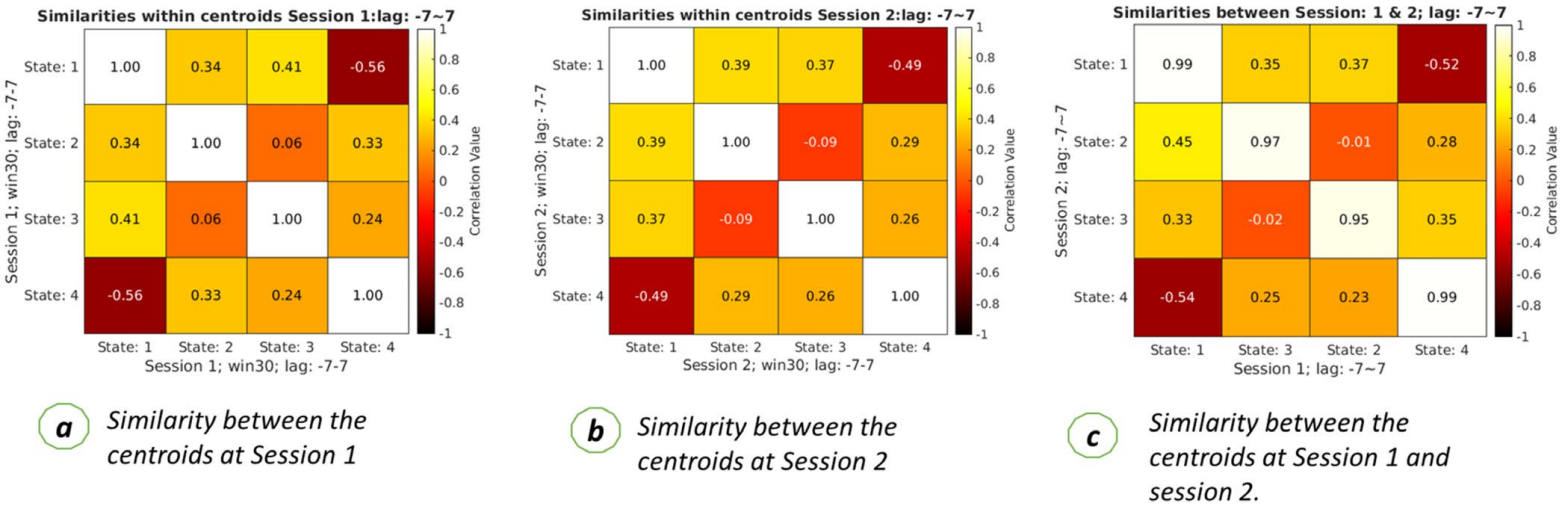
Replicability of results across multiple sessions. The left figure represents the spatial similarity between the centroids or dynamic states calculated from session 1 data. Each cell represents the correlation coefficient between two centroids calculated from clustering. The middle figure represents the spatial similarity between the dynamic states or clustering centroids calculated from session 2 data. The correct figure represents the spatial similarity between the centroids of session 1 and session 2 data. This shows a high similarity between the states between the two sessions, which can be seen from the main diagonal cells. The high correlation value proves that the proposed pipeline produces reproducible centroids over multiple sessions.

In a data‐driven approach, replicability is an important factor both for validity and interpretability (Adali and Calhoun 2022). Replicability ensures that given same subjects with different data produce similar results. The proposed method was tested over two sessions of the HCP‐EP dataset to evaluate replicability. Analysis was run over the same subjects over two different sessions, and the final aggregated centroids, called dynamic states, in two independent analyses. After obtaining two sets of dynamic states, correlation was calculated between the centroids in each session and between sessions. The results show that the dynamic state captured by the two sessions was highly correlated. Figure 2 shows highly replicable results across multiple sessions. Figure 2a represents the spatial similarity between spatial maps in the dynamic states calculated from data using session 1. Cell values represent how similar the spatial maps across the states are calculated from session 1 data only.

Similarly, Figure 2b represents the spatial similarity between the spatial maps at different states computed from session 2 data. Figure 2c represents spatial similarity between the spatial maps located at session 1 against the spatial maps of different states of session 2. After reordering the states based on the maximum similarity, it was observed that spatial maps at state 1 of sessions 1 and 2 were highly similar as they had a high correlation value of 0.99. A similar observation was observed in the case of state 4 of both sessions 1 and 2. Also, spatial maps at state 2 of session 1 were highly similar to spatial maps calculated at state 3 of session 2 with correlation values of 0.95–0.99. The primary diagonal value of Figure 2c shows how the spatial maps at different states are similar. These results also provide evidence that the proposed pipeline provides replicable results.

**FIGURE 3:**
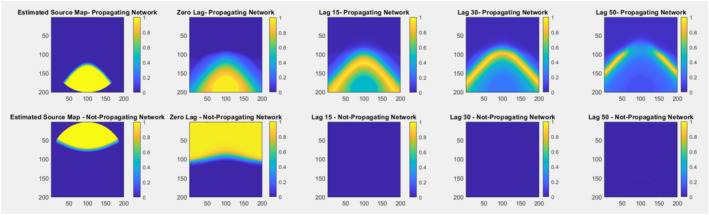
Simulation of network propagation with time shows how network changes as signal propagates.

To illustrate the proposed propagation approach, we designed a simple simulation. Using simulation toolbox (simTB) (Erhardt et al. 2012), we sampled 2D fMRI data consisting of 200 volumes and applied PCA and ICA to generate component maps and time courses. Then, we calculated the lagged correlation between fMRI data and component time courses. The simulation maps are shown in Figure 3. Based on the simulated data, we showed how a network's spatial map behaves as signal propagates over time. In Figure 3, we can see that as signal propagates the spatial maps also change accordingly with respect to the state of the network. The change in correlation value between signal and network changes with time indicates this phenomenon.

**FIGURE 4:**
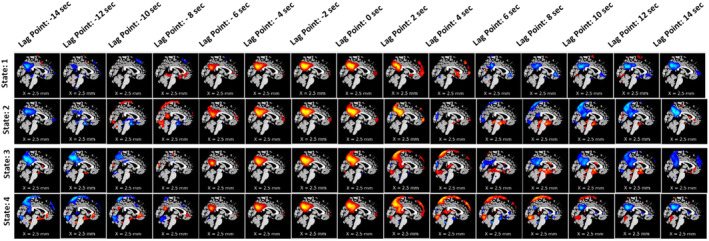
Spatial propagation of the DMN over time. The figure shows how different dynamic states of DMN are fully formed early at a time (lag point: −4 s) and slowly dissipate at lag point 0 s (state 3). Also, the speed of disperses varies from state to state. It also shows that DMN stays positively correlated with the BOLD signal for around 6–8 s (starting from the lag point: −6 s to lag point: 2 s) before it slowly dissolves and becomes anticorrelated.

Next, to observe the spatial propagation of brain networks, we plotted the dynamic spatial maps of the networks. In Figure 4, we plotted the DMN. With our proposed approach, we were able to capture the spatially propagating patterns over time. Figure 4 shows how the dynamic states of the DMN propagate in different states. We also can observe the DMN network remains positively correlated for a certain period before it slowly becomes anticorrelated.

**FIGURE 5:**
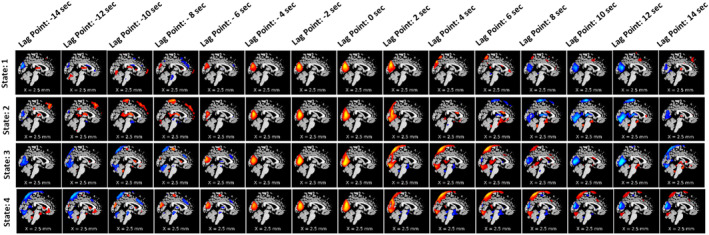
Spatial propagation of the visual network over time. The figure shows how different dynamic states of the visual network are fully formed early (lag point: −4 s) and start to dissipate at lag point 0 s. Also, the speed of disperses varies from state to state. It also shows that the visual network stays positively correlated with the BOLD signal for around 4 s (starting from the lag point: −4 s to lag point: 0 s) before it slowly dissolves and become anticorrelated.

**FIGURE 6:**
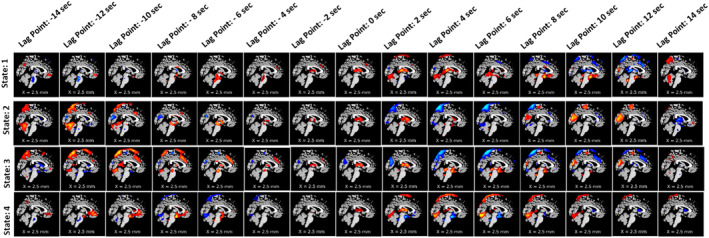
Spatial propagation of the subcortical network over time. The figure shows how different dynamic states of the subcortical network are fully formed at the time (lag point: 0 s) and start to dissipate at lag point 2 s. Also, the speed of disperses varies from state to state. It also shows that the subcortical network stays positively correlated with the BOLD signal for around 2 s (starting from the lag point: 0 s to lag point: 2 s) before it slowly dissolves and becomes anticorrelated.

**FIGURE 7:**
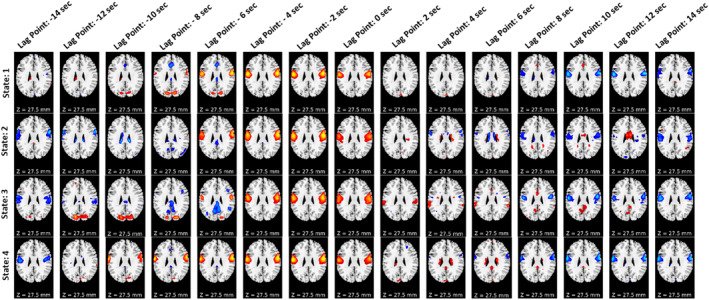
Spatial propagation of the temporal network over time. The figure shows how different dynamic states of temporal network are fully formed early at the time (lag point: −4 s) and start to dissipate quickly at lag point 2 s. Also, the speed of disperses varies from state to state. It also shows that the temporal network stays positively correlated with the BOLD signal for around 4 s (starting from the lag point: −4 s to lag point: 0 s) before it quickly dissolves and becomes anticorrelated.

To observe the spatial propagation of other networks, visual, SC, and temporal were selected. The spatial propagation patterns were observed after running the pipeline independently for each network. Figure 5 shows the visual network, Figure 6 shows the subcortical network, and Figure 7 shows the temporal network. From the figures, it is observed that different networks propagate similarly. However, the propagation rate is different for each network individually.

**FIGURE 8:**
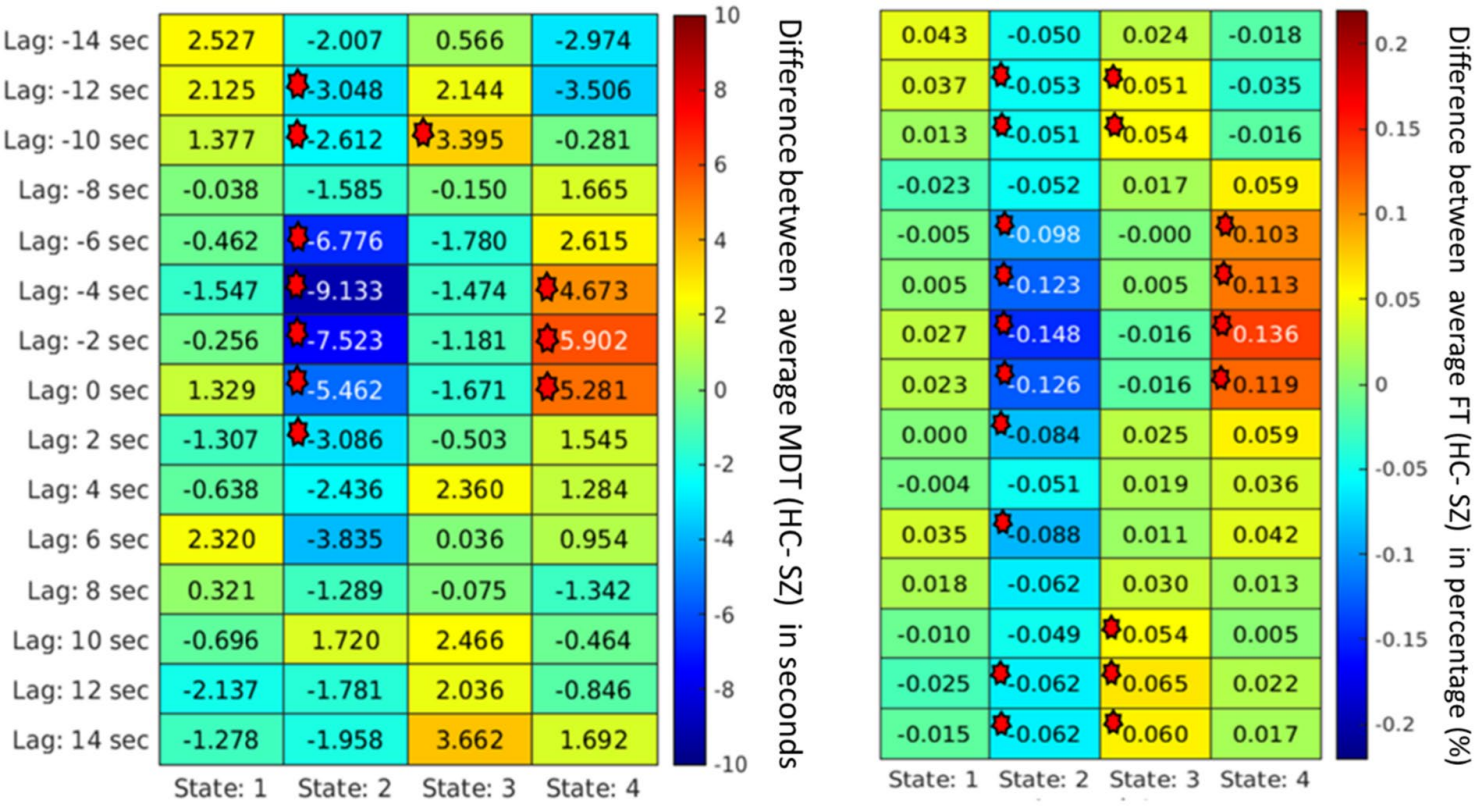
For the visual network, the mean dwell time between two groups captures significant differences at different states at different lags. Each cell value represents the difference between the two groups' average MDT (HC–SZ). Significant differences (*p* < 0.05 (FDR corrected) are marked. The marks are based on corrected *p* value < 0.05 collected form the t‐test between healthy controls and individuals with schizophrenia group. States 2 and 4 captured significant group differences where in state 2, most schizophrenia subjects dwelled longer; in state 4, healthy controls dwelled longer on average in visual network states where schizophrenia patients dwelled more captured significant group differences.

**FIGURE 9:**
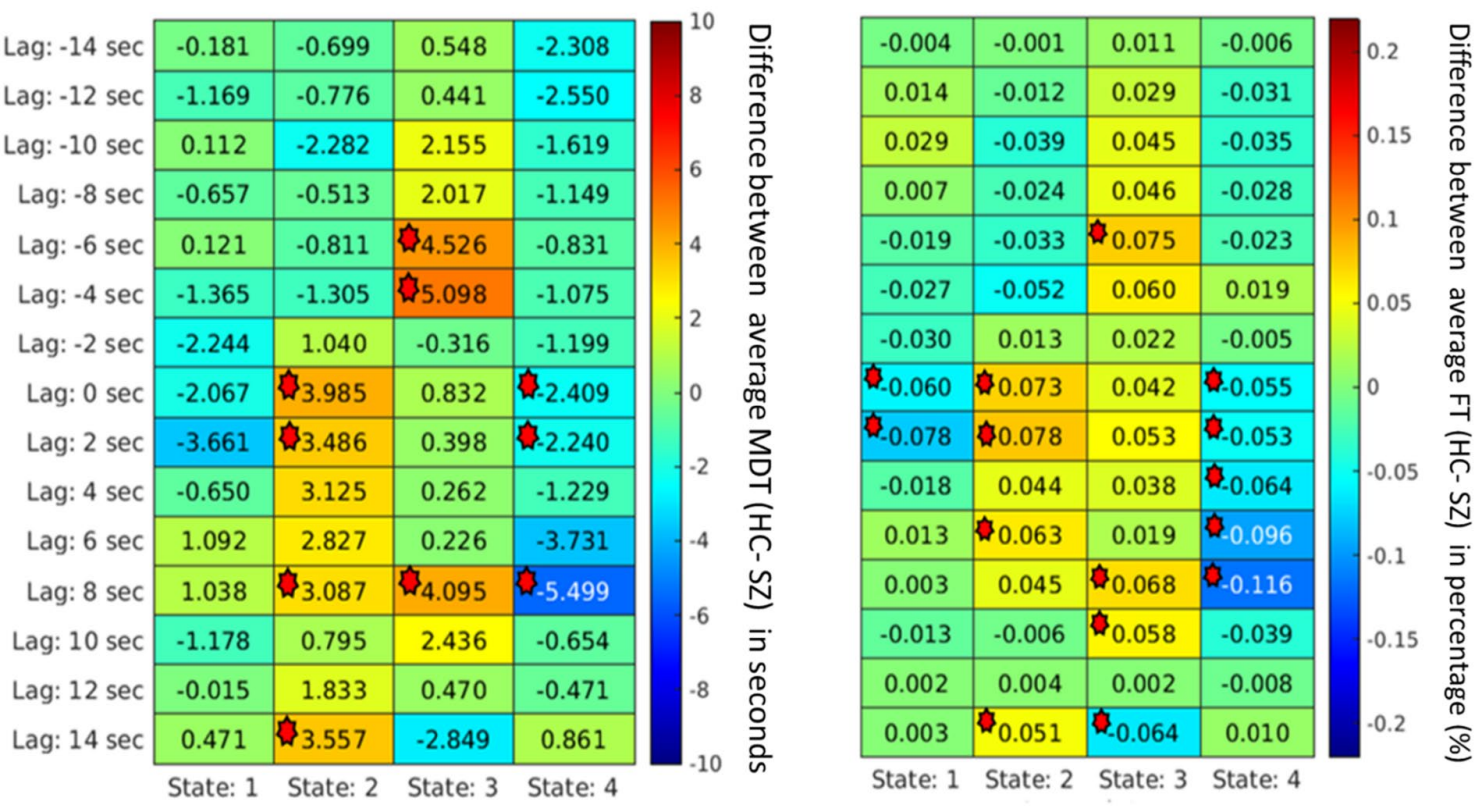
For the default mode network, mean dwell time between two groups captures significant group differences at different states at different lags. Each cell value represents the difference between the two groups' average MDT (HC–SZ). Significant differences (*p* < 0.05) are marked. The marks are based on corrected *p* value < 0.05 collected form the t‐test between healthy controls and individuals with schizophrenia group. The SZ group dwells more in states 1 and 4 than the healthy controls, unlike visual network states where healthy control resides more capture significant group difference.

**FIGURE 10:**
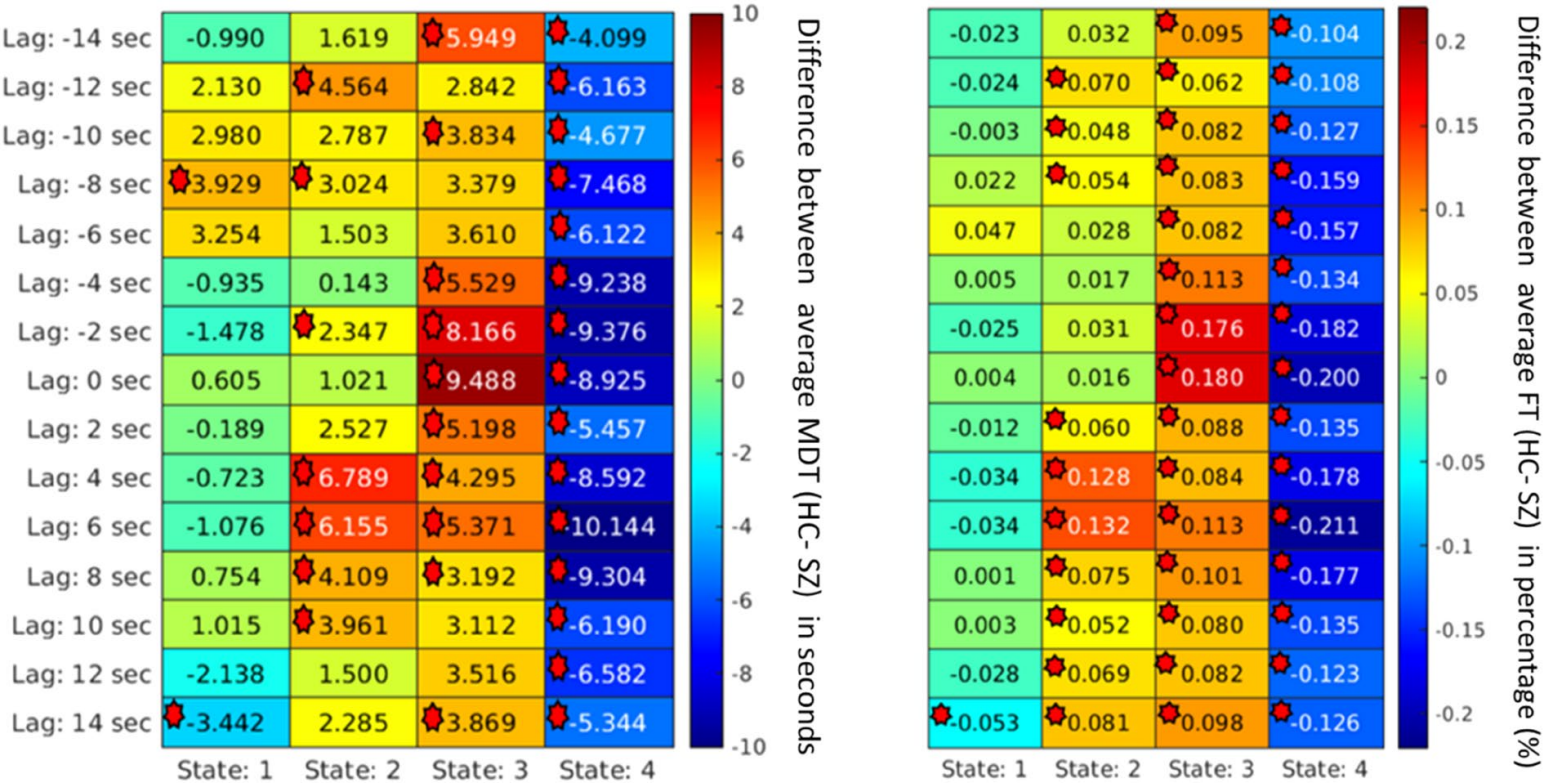
For the subcortical network, the mean dwell time between two groups captures significant group differences at different states at different lags. Each cell value represents the difference between the two groups' average MDT (HC–SZ). Significant differences (*p* < 0.05) are marked. The marks are based on corrected *p* value < 0.05 collected form the t‐test between healthy controls and individuals with schizophrenia group. Most of the schizophrenia group dwelled in most of the time in state 4 over the evolving time. On average, most healthy controls also resided in state 3, suggesting groups do not change conditions between the dynamic and subcortical states.

**FIGURE 11:**
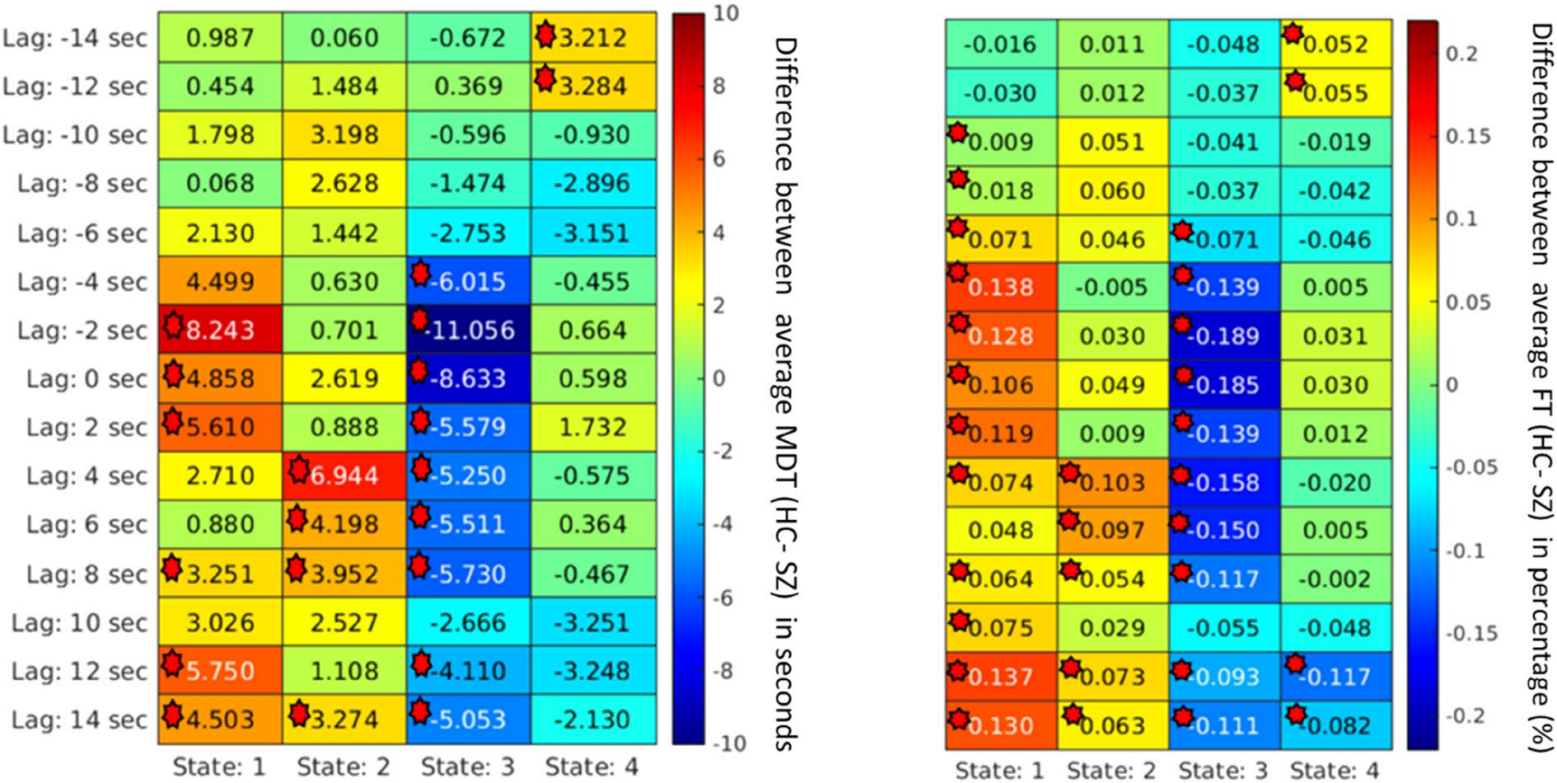
For the temporal network, the mean dwell time between two groups captures significant group differences at different states at different lags. Each cell value represents the difference between the two groups' average MDT (HC–SZ). The marks are based on corrected *p* value < 0.05 collected form the t‐test between healthy controls and individuals with schizophrenia group. Significant differences (*p* < 0.05) are marked. After the network is formed, the schizophrenia group dwells more in a particular state, such as state 3, and healthy control resides more in state 1 most of the time.

To analyze the importance of various spatial patterns that were captured, several measurements, such as MDT, were calculated for each spatial map. First, the group differences based on the MDT at different lags were computed and plotted. It captured group differences at different lag points for different states. In a lag‐less analysis, this information is never captured. Figures 8, 9, 10, 11 plot the average difference of MDT of two groups, and significant states are marked. The marks are based on corrected *p* value < 0.05 collected form the t‐test between HCs and individuals with SZ group. For example, we observed group differences at states 2, 3, and 4 when the lag was at 8 s in DMN in Figure 9. However, only states 2 and 4 show significant group difference at lag 0 s.

Similarly, we can capture group differences for all the states if we observe lag at −8 and −2 s for the SC network shown in Figure 10. Similarly, the FT was computed to capture group differences between the two groups. Figures 8, 9, 10, 11 plot the FT calculated at different lag points for all dynamic states. It was observed that FT at other lag points changes with time as the network propagates with time, and FT changes, too. However, different states located at different lag points capture significant group differences.

Although some significant states are not shown in the figure due to *p* value correction for FDR, Figures 8, 9, 10, 11 capture an overview of different dynamic states, capturing information regarding the group differences related to neurological illness. Results reveal some interesting patterns among the spatial states, such that most group effects are located around the center when the network is more visible, and lag is minimal. Also, it was observed that in some dynamic states, subjects with SZ dwell significantly longer than the healthy control group on average.

## 4 Discussion

The brain is a complex dynamic system. Static analysis of fMRI data may provide important information; however, such research cannot capture the time‐varying behavior of the brain. Recent studies have proposed various ways to capture important aspects of brain dynamism by brain connectivity. Still, these studies ignore the brain's spatial aspects, which evolve with time. The proposed study focused on how each brain network propagates with time over space. The proposed approach showed that spatial information of brain networks changes with time. This study examined four brain networks individually and found that spatial pattern changes as the BOLD signal propagates across the brain. The spatial patterns may differ based on the selected network. However, the observed brain network showed similar propagation behavior. Observing these propagating patterns was the primary goal of this study. There has been little work in this area focused on understanding the dynamics of the propagation of time‐varying states of the brain.

Propagation refers to how neural activity of a network propagated over time causing the changing pattern of the network at the voxel level, which includes, but is not specific to, the borders. The distance between spatial maps at different lags gives us an indirect measurement of the speed of propagation. The changes in the distance values provide an indication of how the spatial maps change over time.

In neuroimaging dynamic analysis, replicability is a considerable concern. In our proposed pipeline, strict correspondence cannot be enforced between different runs due to the data‐driven nature of the approach. For example, ICA may not always produce the same network in the same order. Calculated dynamic states occur in different orders. Despite these challenges, we show that the proposed analysis produces similar states across multiple runs, irrespective of their ordering, in data collected from the same subjects at different times. That supports the hypothesis that the proposed analysis has a high level of replicability.

This study captures how the brain network patterns propagate over time. As the signal travels through the brain, the brain networks interact. From our analysis, we captured dynamic spatial maps and their propagation patterns with respect to time. Each map captures the spatiotemporal correlation values between the ICA time course and the BOLD signals. The maps also represent which brain parts are related to the target network that becomes active with time. For example, from the spatial map propagation maps, we can observe that at different lag points, different brain areas show activation other than the target network, which suggests that brain networks are related and interact with each other as signals travel through time.

In the analysis of the networks, the visual network exhibits the least diversity in state maps. The propagation structures across the four states between lags −4 and +4 are largely similar, with the exception of state 2, which demonstrates a lower correlation beginning at lag 4. Additionally, the propagation maps tend to resemble those observed at lower lag points after lag 8, indicating the emergence of a periodic structure. This periodicity is consistent with the controlled nature of the visual network, particularly under the eyes‐closed condition. Impaired visual processing is a well‐documented issue in SZ, with the visual sensory network (VSN) likely playing a significant role in the disorder (Weinberger 1987). A previous study explored the dynamic functional network connectivity within the VSN, including middle temporal, middle occipital, calcarine, inferior occipital, lingual, fusiform gyri, and cuneus and its association with visual learning in SZ subjects (Sendi, Pearlson, et al. 2021).

The propagation patterns of the SC networks exhibit considerable diversity across different states. As lag increases, the SC networks propagate to various regions. For example, positive correlations with the somatomotor (SM) regions are observed in states 1 and 4, while states 2 and 3 show negative correlations with these regions. Additionally, propagation to visual (VI) areas is evident in both states 1 and 4. Notably, in state 3, the network propagates to frontal regions at lag −10. These findings are consistent with the broad functional roles associated with subcortical networks. A prior study also demonstrated that spontaneous neural activity in subcortical regions can be broken down into multiple independent signals, which correlate with or “echo” the activity of functional networks across the cortex. Specific subregions of the thalamus, striatum, claustrum, and hippocampus exhibited diverse echo patterns from networks involved in attention, control, visual processing, somatomotor functions, and the default mode (Groot et al. 2023). Considering their essential role in functional integration, subcortical regions, are significantly impaired in individuals with SZ (Fan et al. 2019; Yamamoto et al. 2022).

Similarly, in DMN network propagation structures across the four states between lags −6 to 0 are largely similar. In the spatial maps showed spontaneous connections between the DMN, left postcentral gyrus and right postcentral gyrus. The DMN is suggested to play a pivotal role in SZ; previous works also studied the different dynamic patterns and connectivity between the different regions of DMN with respect to the individuals with SZ (Rashid et al. 2019; Hu et al. 2017; Sendi, Zendehrouh, et al. 2021; Wang et al. 2015).

We also observed that the ICA network becomes positively correlated and more visible with time. Most of the significant group differences were captured within such periods. However, in some cases, we also observed that while the network can capture group differences in different dynamic states, it remains visible, although anticorrelated, with respect to the BOLD signal. This suggests that anticorrelated spatial patterns can also capture important neurological information related to illness. Such anticorrelated brain network patterns were captured before in traditional dynamic studies, suggesting that different networks are anticorrelated with respect to temporal correlations (Uddin et al. 2009; Fox et al. 2009). Our study shows that these anticorrelated patterns are time‐dependent. Also, these anticorrelated patterns suggest that the networks do not work independently; instead, different networks share a time‐dependent correlation between them. Previous static analysis did not capture such information.

We also observed that subjects with SZ dwell in a particular state longer before they traverse to the next state. For example, state 3 of temporal, state 3 of visual, state 4 of DMN, and subcortical shows the dwell time of subjects with SZ is larger than that of HC subjects. From the state maps, we also observed how different subnetworks become active with time. For example, different subnetworks of the ICA subcortical network were active at different lag points, and each of them was able to capture significant group differences based on calculated MDT. Because we had a model order of 20, which is considered a lower model order, some of the subnetwork overlapped, and these networks do capture group differences when they become active with time. This explains why we were able to capture group differences when the target network was not very visible.

We also observed that each network has a particular state where SZ group members dwell longer than other states. These states also showed a significant group difference. Networks such as the temporal lobe, visual, and subcortical showed a similar pattern. Previous studies also observed that dwell time in one of the dynamic states was longer than other dynamic states (Adali and Calhoun 2022; Erhardt et al. 2012), which is aligned with our observations.

Furthermore, our results show that lag‐based analysis can capture more states than lag‐less analysis results where no lag is used. These additional lagged states can also capture substantial group differences and convey potentially important and distinct information. Also, it was observed that different states contain different amounts of information. For example, MDT and FT between groups change as the spatial pattern varies with time. This also strengthens our second hypothesis that spatial patterns at different times contain significant information that can be useful for identifying groups and clinical purposes. Such states were ignored in previous studies.

Moreover, temporal embedding transforms time series data into a lower‐dimensional state space, making it invaluable for studying chaotic and nonlinear systems such as time series forecasting, fMRI data analysis, and pattern recognition (Tan et al. 2023). By reconstructing state spaces, it reveals hidden structures, attractors, and periodic behaviors, offering insights into a system's evolution and long‐term dynamics. Our proposed fMRI method builds on these principles, using lagged windowed correlations to track brain network activity over time. These lagged spatial maps serve as embedding dimensions, capturing neural activity's spatial and temporal propagation. Similar to temporal embedding, this approach relies on lagged correlations to uncover dynamic patterns and transitions, balancing temporal resolution to capture meaningful brain network changes.

## 5 Limitations and Future Directions

The results presented in this study have certain limitations. In this study, we used resting‐state eyes‐closed fMRI data. One of the challenges of eyes closed is individuals may sleep more easily. In our case, individuals did indicate they were awake in post‐scan interviews conducted on a random subset of subjects. While this is not definitive, it does provide confidence that they were following instructions. Readers should consider this limitation while interpreting the results.

We only studied four brain networks independently ignoring the presence of internetwork interaction. Another limitation of the current study is that we could not fully understand the fact that whether the observed propagation pattern can be solely attributed to changing neuronal activation, or by the hemodynamic nature of the fMRI BOLD signal. The current study is only the primary step toward understanding the complex nature of dynamics.

In future studies, we will explore these internetwork relationships and how networks relay information. We will also extend the work to extract more features that would help us understand the complexity of spatial propagation patterns.

## 6 Conclusion

This study proposed a novel approach to observe the propagation patterns in the dynamic spatial states of brain networks. The proposed method was tested against data collected from two sessions of the HCP dataset, which showed high replicability. The study focuses on the evolution of spatial patterns of various brain networks that vary over time, which have not been studied previously. These time‐varying spatial networks are statistically significant for group analysis between patients with SZ and controls. In summary, this study can be considered a building block toward thoroughly understanding the complexity of global propagation.

## Author Contributions

**Biozid Bostami:** conceptualization, formal analysis, investigation, methodology, software, visualization, writing – original draft, writing – review and editing. **Noah Lewis:** formal analysis, writing – review and editing. **Oktay Agcaoglu:** formal analysis, investigation, simulation, review and editing. **Jessica A. Turner**, **Theo van Erp**, and **Judith M. Ford:** data collection, writing – review and editing. **Mahshid Fouladivanda:** writing – review and editing. **Vince Calhoun:** conceptualization, funding acquisition, investigation, methodology, project administration, resources, software, writing – review and editing. **Armin Iraji:** conceptualization, formal analysis, writing – review and editing.

## Conflicts of Interest

The authors declare no conflicts of interest.

## Data Availability

The data that support the findings of this study are available on request from the corresponding author. The data are not publicly available due to privacy or ethical restrictions.

## References

[hbm70131-bib-0001] Abbas, A. , Y. Bassil , and S. Keilholz . 2019. “Quasi‐Periodic Patterns of Brain Activity in Individuals With Attention‐Deficit/Hyperactivity Disorder.” NeuroImage: Clinical 21: 101653. 30690417 10.1016/j.nicl.2019.101653 PMC6356002

[hbm70131-bib-0002] Adali, T. L. , and V. D. Calhoun . 2022. “Reproducibility and Replicability in Neuroimaging Data Analysis.” Current Opinion in Neurology 35, no. 4: 475–481. 35856915 10.1097/WCO.0000000000001081 PMC9309985

[hbm70131-bib-0003] Allen, E. A. , E. Damaraju , S. M. Plis , E. B. Erhardt , T. Eichele , and V. D. Calhoun . 2014. “Tracking Whole‐Brain Connectivity Dynamics in the Resting State.” Cerebral Cortex 24, no. 3: 663–676. 23146964 10.1093/cercor/bhs352 PMC3920766

[hbm70131-bib-0004] Allen, E. A. , E. B. Erhardt , E. Damaraju , et al. 2011. “A Baseline for the Multivariate Comparison of Resting‐State Networks.” Frontiers in Systems Neuroscience 5: 2. 21442040 10.3389/fnsys.2011.00002 PMC3051178

[hbm70131-bib-0005] Arthur, D. , and S. Vassilvitskii . 2007. “K‐Means Plus Plus: The Advantages of Careful Seeding.” In Proceedings of the Eighteenth Annual Acm‐Siam Symposium on Discrete Algorithms, 1027–1035. New Orleans, Louisiana: SIAM.

[hbm70131-bib-0006] Biswal, B. , F. Zerrin Yetkin , V. M. Haughton , and J. S. Hyde . 1995. “Functional Connectivity in the Motor Cortex of Resting Human Brain Using Echo‐Planar MRI.” Magnetic Resonance in Medicine 34, no. 4: 537–541. 8524021 10.1002/mrm.1910340409

[hbm70131-bib-0007] Buckner, R. L. , F. M. Krienen , A. Castellanos , J. C. Diaz , and B. T. T. Yeo . 2011. “The Organization of the Human Cerebellum Estimated by Intrinsic Functional Connectivity.” Journal of Neurophysiology 106, no. 5: 2322–2345. 21795627 10.1152/jn.00339.2011 PMC3214121

[hbm70131-bib-0008] Calhoun, V. D. , T. Adali , G. D. Pearlson , and J. J. Pekar . 2001. “Spatial and Temporal Independent Component Analysis of Functional MRI Data Containing a Pair of Task‐Related Waveforms.” Human Brain Mapping 13, no. 1: 43–53. 11284046 10.1002/hbm.1024 PMC6871956

[hbm70131-bib-0009] Calhoun, V. D. , K. A. Kiehl , and G. D. Pearlson . 2008. “Modulation of Temporally Coherent Brain Networks Estimated Using ICA at Rest and During Cognitive Tasks.” Human Brain Mapping 29, no. 7: 828–838. 18438867 10.1002/hbm.20581 PMC2649823

[hbm70131-bib-0010] Calhoun, V. D. , R. Miller , G. Pearlson , and T. Adalı . 2014. “The Chronnectome: Time‐Varying Connectivity Networks as the Next Frontier in fMRI Data Discovery.” Neuron 84, no. 2: 262–274. 25374354 10.1016/j.neuron.2014.10.015 PMC4372723

[hbm70131-bib-0011] Cox, R. W. 1996. “AFNI: Software for Analysis and Visualization of Functional Magnetic Resonance Neuroimages.” Computers and Biomedical Research 29, no. 3: 162–173. 8812068 10.1006/cbmr.1996.0014

[hbm70131-bib-0012] Damaraju, E. , E. A. Allen , A. Belger , et al. 2014. “Dynamic Functional Connectivity Analysis Reveals Transient States of Dysconnectivity in Schizophrenia.” Neuroimage‐Clinical 5: 298–308. 25161896 10.1016/j.nicl.2014.07.003 PMC4141977

[hbm70131-bib-0013] Du, Y. , and Y. Fan . 2013. “Group Information Guided ICA for fMRI Data Analysis.” NeuroImage 69: 157–197. 23194820 10.1016/j.neuroimage.2012.11.008

[hbm70131-bib-0014] Erhardt, E. B. , E. A. Allen , Y. Wei , T. Eichele , and V. D. Calhoun . 2012. “SimTB, a Simulation Toolbox for fMRI Data Under a Model of Spatiotemporal Separability.” NeuroImage 59, no. 4: 4160–4167. 22178299 10.1016/j.neuroimage.2011.11.088 PMC3690331

[hbm70131-bib-0015] Fan, F. , H. Xiang , S. Tan , et al. 2019. “Subcortical Structures and Cognitive Dysfunction in First Episode Schizophrenia.” Psychiatry Research: Neuroimaging 286: 69–75. 30921760 10.1016/j.pscychresns.2019.01.003 PMC6475899

[hbm70131-bib-0016] Fox, M. D. , and M. E. Raichle . 2007. “Spontaneous Fluctuations in Brain Activity Observed With Functional Magnetic Resonance Imaging.” Nature Reviews. Neuroscience 8, no. 9: 700–711. 17704812 10.1038/nrn2201

[hbm70131-bib-0017] Fox, M. D. , D. Zhang , A. Z. Snyder , and M. E. Raichle . 2009. “The Global Signal and Observed Anticorrelated Resting State Brain Networks.” Journal of Neurophysiology 101, no. 6: 3270–3283. 19339462 10.1152/jn.90777.2008 PMC2694109

[hbm70131-bib-0018] Fu, Z. , A. Iraji , J. Sui , and V. D. Calhoun . 2021. “Whole‐Brain Functional Network Connectivity Abnormalities in Affective and Non‐Affective Early Phase Psychosis.” Frontiers in Neuroscience 15: 682110. 34220438 10.3389/fnins.2021.682110 PMC8250435

[hbm70131-bib-0019] Fu, Z. N. , A. Iraji , J. A. Turner , et al. 2021. “Dynamic State With Covarying Brain Activity‐Connectivity: On the Pathophysiology of Schizophrenia.” NeuroImage 224: 117385. 32950691 10.1016/j.neuroimage.2020.117385 PMC7781150

[hbm70131-bib-0020] Groot, J. M. , S. Miletic , S. J. S. Isherwood , et al. 2023. “Echoes From Intrinsic Connectivity Networks in the Subcortex.” Journal of Neuroscience 43, no. 39: 6609–6618. 37562962 10.1523/JNEUROSCI.1020-23.2023 PMC10538587

[hbm70131-bib-0021] “Human Coonectome Dataset.” http://www.humanconnectomeproject.org/data/.

[hbm70131-bib-0022] “Gift Toolbox for ICA.” https://trendscenter.org/software/gift/.

[hbm70131-bib-0023] “SPM12.” https://www.fil.ion.ucl.ac.uk/spm/software/spm12/.

[hbm70131-bib-0024] Hu, M. L. , X. F. Zong , J. J. Mann , et al. 2017. “A Review of the Functional and Anatomical Default Mode Network in Schizophrenia.” Neuroscience Bulletin 33, no. 1: 73–84. 27995564 10.1007/s12264-016-0090-1 PMC5567552

[hbm70131-bib-0025] Ikegaya, Y. , G. Aaron , R. Cossart , et al. 2004. “Synfire Chains and Cortical Songs: Temporal Modules of Cortical Activity.” Science 304, no. 5670: 559–564. 15105494 10.1126/science.1093173

[hbm70131-bib-0026] Iraji, A. , A. Faghiri , Z. Fu , et al. 2022. “Moving Beyond the ‘CAP’ of the Iceberg: Intrinsic Connectivity Networks in fMRI Are Continuously Engaging and Overlapping.” NeuroImage 251: 119013. 35189361 10.1016/j.neuroimage.2022.119013 PMC9107614

[hbm70131-bib-0027] Iraji, A. , Z. Fu , E. Damaraju , et al. 2019. “Spatial Dynamics Within and Between Brain Functional Domains: A Hierarchical Approach to Study Time‐Varying Brain Function.” Human Brain Mapping 40, no. 6: 1969–1986. 30588687 10.1002/hbm.24505 PMC6692083

[hbm70131-bib-0028] Keator, D. B. , T. van Erp , J. A. Turner , et al. 2016. “The Function Biomedical Informatics Research Network Data Repository.” NeuroImage 124: 1074–1079. 26364863 10.1016/j.neuroimage.2015.09.003 PMC4651841

[hbm70131-bib-0029] Kiviniemi, V. , T. Vire , J. Remes , et al. 2011. “A Sliding Time‐Window ICA Reveals Spatial Variability of the Default Mode Network in Time.” Brain Connectivity 1, no. 4: 339–347. 22432423 10.1089/brain.2011.0036

[hbm70131-bib-0030] Kucyi, A. , A. Tambini , S. Sadaghiani , S. Keilholz , and J. R. Cohen . 2018. “Spontaneous Cognitive Processes and the Behavioral Validation of Time‐Varying Brain Connectivity.” Network Neuroscience 2, no. 4: 397–417. 30465033 10.1162/netn_a_00037 PMC6195165

[hbm70131-bib-0031] Liu, X. , and J. H. Duyn . 2013. “Time‐Varying Functional Network Information Extracted From Brief Instances of Spontaneous Brain Activity.” Proceedings of the National Academy of Sciences of the United States of America 110, no. 11: 4392–4397. 23440216 10.1073/pnas.1216856110 PMC3600481

[hbm70131-bib-0032] Majeed, W. , M. Magnuson , and S. D. Keilholz . 2009. “Spatiotemporal Dynamics of Low Frequency Fluctuations in BOLD fMRI of the Rat.” Journal of Magnetic Resonance Imaging 30, no. 2: 384–393. 19629982 10.1002/jmri.21848 PMC2758521

[hbm70131-bib-0033] Matsui, T. , T. Murakami , and K. Ohki . 2016. “Transient Neuronal Coactivations Embedded in Globally Propagating Waves Underlie Resting‐State Functional Connectivity.” Proceedings of the National Academy of Sciences of the United States of America 113, no. 23: 6556–6561. 27185944 10.1073/pnas.1521299113 PMC4988587

[hbm70131-bib-0034] McKeown, M. J. , L. K. Hansen , and T. J. Sejnowski . 2003. “Independent Component Analysis of Functional MRI: What Is Signal and What Is Noise?” Current Opinion in Neurobiology 13, no. 5: 620–629. 14630228 10.1016/j.conb.2003.09.012 PMC2925426

[hbm70131-bib-0035] Miller, R. L. , G. Pearlson , and V. D. Calhoun . 2019. “Whole Brain Polarity Regime Dynamics Are Significantly Disrupted in Schizophrenia and Correlate Strongly With Network Connectivity Measures.” PLoS One 14, no. 12: e0224744. 31825974 10.1371/journal.pone.0224744 PMC6905532

[hbm70131-bib-0036] Miller, R. L. , V. M. Vergara , G. D. Pearlson , and V. D. Calhoun . 2022. “Multiframe Evolving Dynamic Functional Connectivity (EVOdFNC): A Method for Constructing and Investigating Functional Brain Motifs.” Frontiers in Neuroscience 16: 770468. 35516809 10.3389/fnins.2022.770468 PMC9063321

[hbm70131-bib-0037] Mitra, A. , A. Z. Snyder , T. Blazey , and M. E. Raichle . 2015. “Lag Threads Organize the Brain's Intrinsic Activity.” Proceedings of the National Academy of Sciences of the United States of America 112, no. 17: E2235–E2244. 25825720 10.1073/pnas.1503960112 PMC4418865

[hbm70131-bib-0038] Petersen, C. C. , T. T. Hahn , M. Mehta , A. Grinvald , and B. Sakmann . 2003. “Interaction of Sensory Responses With Spontaneous Depolarization in Layer 2/3 Barrel Cortex.” Proceedings of the National Academy of Sciences of the United States of America 100, no. 23: 13638–13643. 14595013 10.1073/pnas.2235811100 PMC263866

[hbm70131-bib-0039] Rashid, B. , S. I. Dev , M. Esterman , et al. 2019. “Aberrant Patterns of Default‐Mode Network Functional Connectivity Associated With Metabolic Syndrome: A Resting‐State Study.” Brain and Behavior: A Cognitive Neuroscience Perspective 9, no. 12: e01333. 10.1002/brb3.1333 PMC6908882 31568716

[hbm70131-bib-0040] Sendi, M. S. E. , G. D. Pearlson , D. H. Mathalon , et al. 2021. “Multiple Overlapping Dynamic Patterns of the Visual Sensory Network in Schizophrenia.” Schizophrenia Research 228: 103–111. 33434723 10.1016/j.schres.2020.11.055

[hbm70131-bib-0041] Sendi, M. S. E. , E. Zendehrouh , C. A. Ellis , et al. 2021. “Aberrant Dynamic Functional Connectivity of Default Mode Network in Schizophrenia and Links to Symptom Severity.” Frontiers in Neural Circuits 15: 649417. 33815070 10.3389/fncir.2021.649417 PMC8013735

[hbm70131-bib-0042] Stroh, A. , H. Adelsberger , A. Groh , et al. 2013. “Making Waves: Initiation and Propagation of Corticothalamic Ca2+ Waves In Vivo.” Neuron 77, no. 6: 1136–1150. 23522048 10.1016/j.neuron.2013.01.031

[hbm70131-bib-0043] Tan, E. , S. Algar , D. Corrêa , M. Small , T. Stemler , and D. Walker . 2023. “Selecting Embedding Delays: An Overview of Embedding Techniques and a New Method Using Persistent Homology.” Chaos 33, no. 3: 032101. 37003815 10.1063/5.0137223

[hbm70131-bib-0044] Uddin, L. Q. , A. M. Clare Kelly , B. B. Biswal , F. Xavier Castellanos , and M. P. Milham . 2009. “Functional Connectivity of Default Mode Network Components: Correlation, Anticorrelation, and Causality.” Human Brain Mapping 30, no. 2: 625–637. 18219617 10.1002/hbm.20531 PMC3654104

[hbm70131-bib-0045] Wang, H. , L. L. Zeng , Y. Chen , H. Yin , Q. Tan , and D. Hu . 2015. “Evidence of a Dissociation Pattern in Default Mode Subnetwork Functional Connectivity in Schizophrenia.” Scientific Reports 5: 14655. 26419213 10.1038/srep14655 PMC4588504

[hbm70131-bib-0046] Weinberger, D. R. 1987. “Implications of Normal Brain Development for the Pathogenesis of Schizophrenia.” Archives of General Psychiatry 44, no. 7: 660–669. 3606332 10.1001/archpsyc.1987.01800190080012

[hbm70131-bib-0047] Xu, N. , D. M. Smith , G. Jeno , D. T. Seeburger , E. H. Schumacher , and S. D. Keilholz . 2023. “The Interaction Between Random and Systematic Visual Stimulation and Infraslow Quasiperiodic Spatiotemporal Patterns of Whole Brain Activity (Withdrawal of Vol 276, Art no 120165, 2023).” NeuroImage 276: 120165. 37172663 10.1016/j.neuroimage.2023.120165

[hbm70131-bib-0048] Yamamoto, M. , E. Bagarinao , M. Shimamoto , T. Iidaka , and N. Ozaki . 2022. “Involvement of Cerebellar and Subcortical Connector Hubs in Schizophrenia.” Neuroimage Clin 35: 103140. 36002971 10.1016/j.nicl.2022.103140 PMC9421528

